# Dexmedetomidine reduces the inflammation level and morality in adult sepsis: a systemic review and meta-analysis based on randomized controlled trials

**DOI:** 10.3389/fmed.2025.1695924

**Published:** 2025-10-21

**Authors:** Biao Peng, Xuepei Huang, Qiting Xue, Jianming Tang, Fang Wan, Yuyao Peng, Guangjun Jiang, Bo Zhou

**Affiliations:** ^1^Department of Critical Care Medicine, Hunan Want Want Hospital, Changsha, China; ^2^Department of Intensive Care Unit, Yiwu Central Hospital, Yiwu, Zhejiang, China; ^3^Department of Emergency Medicine, The Second People’s Hospital of Changsha County, Changsha, China

**Keywords:** dexmedetomidine, sedative, sepsis, bloodstream infection, shock, meta-analysis

## Abstract

**Background:**

Sepsis is a systemic inflammatory response syndrome characterized by an inflammatory cytokine storm and immune dysregulation. The clinical benefits of dexmedetomidine in patients with sepsis remain unclear. This study aimed to explore the effects of dexmedetomidine on the inflammatory status and clinical outcomes of patients with sepsis.

**Methods:**

This study searched PubMed, Embase, and the Cochrane Library for records from the setup day of each database up to August 1, 2025. The search strategy was as follows: (Dexmedetomidine OR Dexmedetomidine Hydrochloride OR Precedex OR Igami) AND (Sepsis OR Bloodstream Infection OR Bloodstream Infections OR Septicemia OR Septicemias). The primary outcomes included interleukin-6 (IL-6), tumor necrosis factor-*α* (TNF-α), and C-reactive protein (CRP). The secondary outcome measures included in-hospital mortality, ICU mortality, 28-day mortality, length of ICU stay, ventilator-free days at day 28, Sequential Organ Failure Assessment (SOFA) score, and Acute Physiology and Chronic Health Evaluation II (APACHE II) score. Stata 14.0 was used for data analysis.

**Results:**

A total of 1,550 sepsis patients were included in this study, among whom 759 received dexmedetomidine treatment. Regarding inflammatory factors, the analysis results showed that dexmedetomidine significantly reduced interleukin-6 (IL-6) [standardized mean difference (SMD) = 0.04, 95% confidence interval (95%CI) = (−0.11, 0.19), *p* = 0.574] and tumor necrosis factor-*α* (TNF-α) levels [SMD = −2.39, 95, 95%CI = (−3.52, −1.27), *p* < 0.001] in sepsis patients, while exerting no effect on C-reactive protein (CRP) levels. In terms of clinical prognosis, the analysis indicated that dexmedetomidine significantly decreased hospital mortality [relative risk (RR) = 0.65, 95% confidence interval (95%CI) = (0.45, 0.94), *p* = 0.021] and 28-day mortality [RR = 0.68, 95%CI = (0.55, 0.84), *p* < 0.001] in sepsis patients, with no impact on other secondary outcome measures.

**Conclusion:**

Dexmedetomidine can reduce the levels of IL-6 and TNF-αin patients with sepsis, while also decreasing in-hospital mortality and 28-day mortality. Furthermore, early identification of sepsis and subsequent administration of dexmedetomidine for sedation and anti-inflammatory therapy may yield more pronounced clinical benefits.

## 1 Introduction

Sepsis is a systemic inflammatory response syndrome triggered by pathogenic infections, characterized by the formation of an inflammatory factor storm and immune regulation imbalance in the body (1). Chills, fever, palpitation and shortness of breath are the early non-specific clinical manifestations of sepsis. The disease progresses extremely rapidly, and changes in consciousness and hemodynamic instability may occur during the progression of the disease, with an extremely high mortality rate. Previous studies (2) have reported that there were approximately 48.9 million cases of sepsis worldwide in 2017, among which 11 million people died, accounting for about one-fifth of the global total deaths. Developed countries have more advanced medical technologies, but like developing countries, they still face the significant economic burden posed by sepsis. According to statistics from the U.S. Centers for Medicare & Medicaid Services (3), total hospitalization expenditures for sepsis in the United States increased from 17.8 billion U.S. dollars in 2012 to 22.4 billion in 2018. Additionally, nursing costs within 90 days after discharge rose from 3.9 billion to 5.6 billion U.S. dollars over the same period. Therefore, exploring more beneficial intervention measures for sepsis patients is of great clinical significance.

Dexmedetomidine is a highly selective *α*₂-adrenergic receptor agonist that has been approved for clinical use and is widely applied in intensive care units (ICUs) (4). Similar to midazolam and propofol, it exhibits excellent sedative and analgesic effects, while having a lower incidence of delirium (5). Mechanistically, dexmedetomidine exerts its sedative effect by selectively activating *α*₂ receptors in the locus coeruleus of the brainstem. This activation promotes potassium ion efflux, inhibits calcium ion efflux, impairs the firing activity of neurons, and thereby induces endogenous non-rapid eye movement (NREM) sleep (6). Meanwhile, dexmedetomidine potentially plays an important role in anti-inflammation and antioxidant defense in the body. Xiao S and his colleagues (7) observed that in Th2-mediated asthmatic mice treated with dexmedetomidine, there was a significant reduction in eosinophilic airway inflammation, mucus secretion levels, airway hyperresponsiveness, and the levels of Th2 cytokines. Another meta-analysis found that dexmedetomidine could decrease the levels of CRP, IL-6, and TNF-*α* in perioperative patients, accompanied by an increase in natural killer (NK) cells and CD4^+^T cells (8).

Severe sepsis can trigger excessive stress responses in the body, characterized by sympathetic nerve excitation, hypermetabolism, and a hypercatabolic state. This reaction further increases the burden on organs such as the heart, liver, and kidneys (9), with the pathological process involving multiple complex molecular and cellular mechanisms. At the cellular and molecular level, the macrophage pyroptosis microvesicle pathway is a key driver of sepsis progression, which can induce tissue damage, neutrophil extracellular traps formation, and abnormal activation of coagulation processes, thereby exacerbating organ injury (10). Specifically, in lung tissue, ADAR1 exerts a protective effect by regulating the miR-21/A20/NLRP3 axis, which can alleviate sepsis-induced lung injury and hinder the activation of pyroptosis in pulmonary macrophages (11). In cardiac tissue, VDAC2 malonylation participates in sepsis-induced myocardial dysfunction through mitochondria-related ferroptosis (12). In renal tissue, histone H3K18 and Ezrin lactylation can promote renal insufficiency in sepsis-related acute kidney injury. Sepsis is also a high-risk factor for the development of delirium (13). Hence, the administration of sedative drugs is beneficial for patients with sepsis (14). However, the efficacy of dexmedetomidine, a classic sedative, in sepsis patients remains controversial. A randomized controlled trial (RCT) study from Switzerland (15) showed that among sepsis patients in the ICU requiring mechanical ventilation, dexmedetomidine did not exhibit significant differences in markers including S100-*β* and neuron-specific enolase compared with propofol and midazolam. In contrast, another meta-analysis based on 19 RCTs (16) found that dexmedetomidine significantly reduced the levels of S100-β [standardized mean difference (SMD) = −2.73, 95% confidence interval (CI) (−3.65, −1.82)] and NSE [SMD = −1.69, 95% CI (−2.77, −0.61)], accompanied by a decrease in inflammatory mediators. It is worth noting that S100-*β* and NSE are important indicators for predicting the severity of craniocerebral injury (17). Apart from sedative intervention, targeted regulation of pathological mechanisms also shows potential in sepsis management. For example, lysosomal nanoreactors rich in zinc and calcium can rescue monocyte/macrophage dysfunction under sepsis, which may improve the body’s immune regulation ability (18). Additionally, BCG-derived outer membrane vesicles can induce TLR2-dependent trained immunity to prevent polymicrobial sepsis, providing new ideas for the prevention and treatment of sepsis (19).

To further explore the effects of dexmedetomidine on inflammatory levels and survival prognosis in patients with sepsis, we conducted this meta-analysis by searching the PubMed, Embase, and Cochrane databases and collecting randomized controlled blinded studies.

## 2 Methods

### 2.1 Search strategy

In this study, three main databases (PubMed, Embase, and Cochrane Library) were systematically searched to collect relevant research published from inception to August 1, 2025. Terms related to sepsis: Sepsis, Bloodstream Infection, Bloodstream Infections, Septicemia, Septicemias; Terms related to dexmedetomidine: Dexmedetomidine, Dexmedetomidine Hydrochloride. The search strategy was as follows: (Dexmedetomidine OR Dexmedetomidine Hydrochloride OR Precedex OR Igami) AND (Sepsis OR Bloodstream Infection OR Bloodstream Infections OR Septicemia OR Septicemias). All retrieved literature was uniformly managed using EndNote X8 (Thomson Scientific, Canada) software, including literature deduplication, screening and marking, and data organization.

### 2.2 Inclusion and exclusion criteria

Studies included in this meta-analysis must meet the following criteria: (a). Study type match RCT; (b). Study participants: Patients diagnosed with sepsis either empirically by physicians or in accordance with relevant guidelines; (c). Age of participants ≥ 18 years old; (d). Experimental group: Received dexmedetomidine intervention; (e). Outcome measures: The study must include at least one primary outcome measure or secondary outcome measure.

Studies will be excluded if they meet any of the following conditions: (a). Study types mismatch RCT, such as reviews, letters, basic research, and meta-analyses; (b). Age of participants < 18 years old; (c). Duplicate literature retrieved from the three database; (d). Content irrelevant to the topic of this study; (e). Missing data; (f). Unavailable article; (g). Lack of observed outcomes; (h). insufficient scientific rigor of the study.

### 2.3 Outcome measures

The outcome measures included in this study are divided into primary outcome measures and secondary outcome measures. The primary outcome measures include the levels of CRP, IL-6, and TNF-*α* in the peripheral blood of patients with sepsis. The secondary outcome measures include the SOFA score, APACHE II score, length of ICU stay, and 28-day mortality of patients with sepsis.

### 2.4 Data extraction

Two researchers independently extracted data from the included studies. Briefly, for continuous outcome variables, the mean, standard deviation (SD), and sample size of each group were extracted; for binary outcome variables, the number of events and sample size of each group were extracted. If there were discrepancies in the data extracted by the two researchers, data extraction was repeated, and the data was verified by a third researcher. Since some studies only provided the median and interquartile range (IQR) for continuous outcome variables, these were estimated using the calculation methods proposed by Luo (20) and Wan (21).

### 2.5 Quality assessment

In accordance with the criteria of the Grading of Recommendations Assessment, Development, and Evaluation (GRADE), the quality evaluation of each study was conducted using RevMan 5 software downloaded from the Cochrane Community (www.cochrane.org/learn/courses-and-resources/software). Specifically, the included studies were evaluated across 6 domains: selection bias, performance bias, detection bias, attrition bias, reporting bias, and other bias. Each item was independently assessed and categorized as low risk of bias, unclear risk of bias, or high risk of bias.

### 2.6 Statistical analysis

Heterogeneity testing was performed to quantify the extent of variability across individual studies or samples, utilizing both the I^2^ statistic and Cochrane’s Q test. Significant heterogeneity was defined as an I^2^ value > 50% or a *p* value < 0.05, in which case a randomized-effects model was employed. For effect size pooling under this scenario, DerSimonian-Laird (D-L) method was applied. Conversely, in the absence of heterogeneity, either the Mantel–Haenszel (M-H) method or the inverse variance method was applied to pool effect sizes under the fixed-effects model. The pooled effect size was expressed as an risk ratio (RR) or standard mean difference (SMD). Sensitivity analysis was performed to evaluate the stability of the results. All statistical analyses were performed using Stata 14.0 software (StataCorp LLC, United States).

## 3 Result

### 3.1 Search results

**Figure 1:**
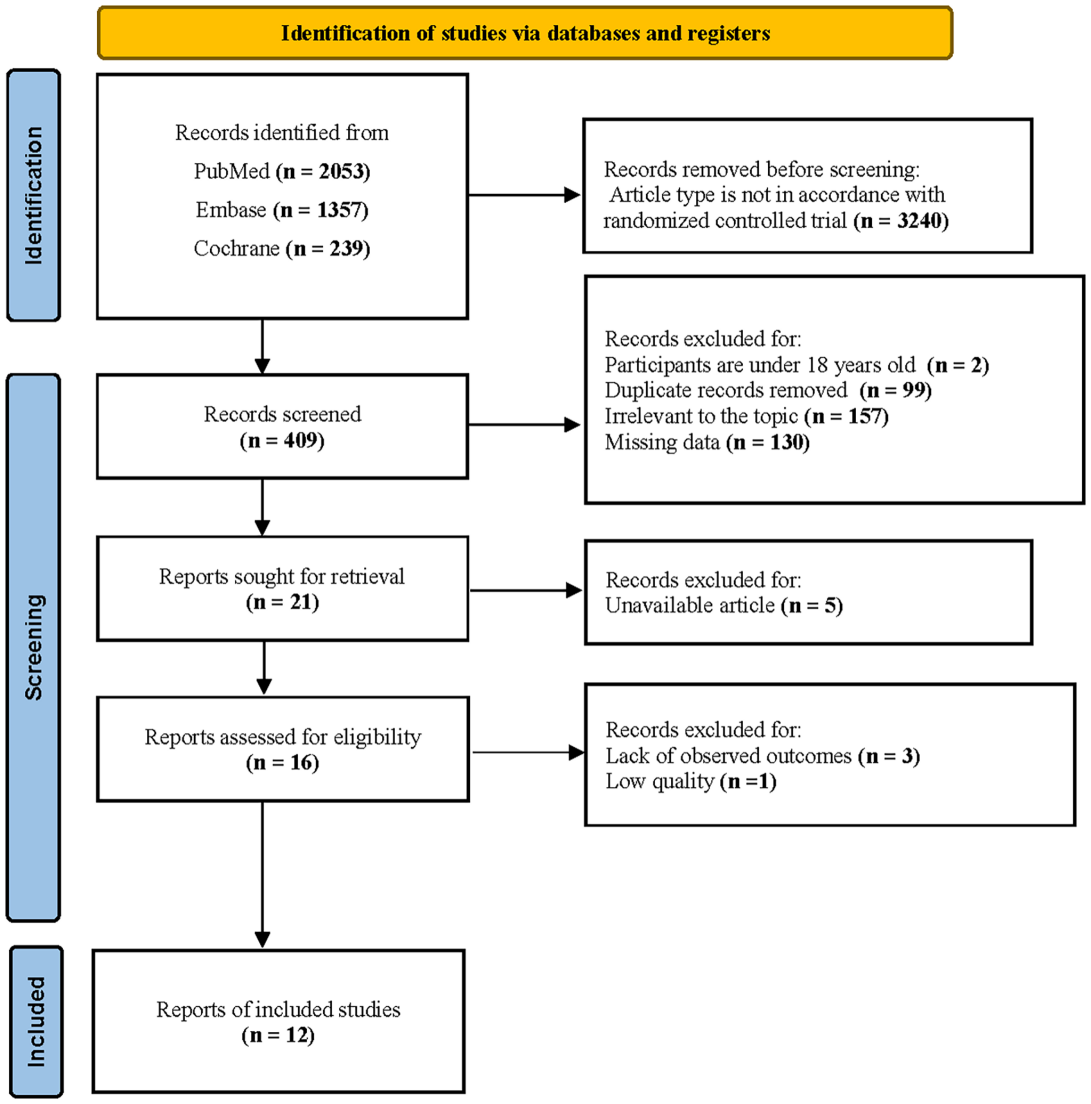
Literature screening flow diagram.

A total of 3,649 records were retrieved from three major databases, with 2,053 from PubMed, 1,357 from Embase, and 239 from the Cochrane Library. After screening, 3,240 studies were excluded for not meeting the RCT criteria, 2 studies were excluded because their participants were under 18 years old, 99 were removed as duplicate records, 157 were excluded for being irrelevant to the research topic, 130 were excluded due to unavailable data, 5 were inaccessible, 3 lacked outcome measures, and 1 was excluded due to insufficient scientific rigor of the study. Finally, 12 studies were included in this study (Figure 1).

### 3.2 Study characteristics and quality

The included studies were published between 2009 and 2025, all of which were RCTs published in English. The smallest sample size was 32, while the largest was 442, and all participants were over 18 years old. Geographically, 3 studies were conducted in Japan, 2 in the United States, 2 in Egypt, 1 in China, 1 in France, 1 in Türkiye, 1 in Iran, and 1 was a collaborative study by international organizations. Differences existed in the diagnostic criteria for sepsis and the maintenance dose of dexmedetomidine across the included studies, with specific details shown in Table 1.

**Table 1:** The characteristics of included studies.

Author (year)	Study type	Sample Size		Age (years)		Country/Region	Diagnostic criteria for sepsis	Maintenance dose (μg/kg/h)
		Dex group	Cont group	Dex group	Cont group			
Ezz Al-Re (2024) (39)	RCT	45	45	59 ± 16.6	61 ± 14	Egypt	SCCM/ESICM 2016 (Sepsis-3) (40)	0.7–1.0
Chen (2018) (41)	RCT	80	80	46.41 ± 5.95	47.57 ± 4.48	China	SCCM/ESICM 2016 (Sepsis-3)	0.2–0.7
Dargent (2025) (22)	RCT	16	16	62.2 ± 16.1	67.1 ± 11.2	France	SCCM/ESICM 2016 (Sepsis-3)	0.7–1.0
Hughes (2021) (42)	RCT	214	208	59 (48–68)	60 (50–68)	United States	SCCM/ESICM 2016 (Sepsis-3)	0.2–1.5
Moore (2022) (43)	RCT	51	52	61 ± 15.9	66.6 ± 10.5	International group	SCCM/ESICM 2016 (Sepsis-3)	Not disclosed
Miyamoto (2018) (44)	RCT	60	51	70.0 ± 14.3	72.1 ± 12.3	Japan	ACCP/SCCM 1992 (45)	Not disclosed
Mokhlesian (2025) (46)	RCT	24	24	48.15 ± 8.19	50.29 ± 8.55	Egypt	SCCM/ESICM 2016 (Sepsis-3)	0.2–0.7
Mohamed (2022) (47)	RCT	64	64	61.67 ± 16.44	59.58 ± 18.34	Iran	SCCM/ESICM 2016 (Sepsis-3)	0.2–2.5
Tasdogan (2009) (48)	RCT	20	20	50 (19–74)	58 (21–78)	Türkiye	ACCP/SCCM 1992	0.2–2.5
Pandharipande (2010) (49)	RCT	31	32	60 (46–65)	58 (44–66)	United States	ACCP/SCCM 1992	0.2–1.5
Nakashima (2020) (23)	RCT	54	50	70.7 ± 15.1	71.4 ± 13.2	Japan	ACCP/SCCM 1992	Not disclose
Kawazoe (2017) (50)	RCT	100	101	68 ± 14.9	69 ± 13.6	Japan	ACCP/SCCM 1992	Not disclose

RCT, randomized controlled trial; Dex group, dexmedetomidine group; Cont group, control group. SCCM/ESICM 2016, the Society of Critical Care Medicine and European Society of Intensive Care Medicine 2016 (Sepsis-3); ACCP/SCCM 1992, American College of Chest Physicians/Society of Critical Care Medicine 1992.

**Figure 2:**
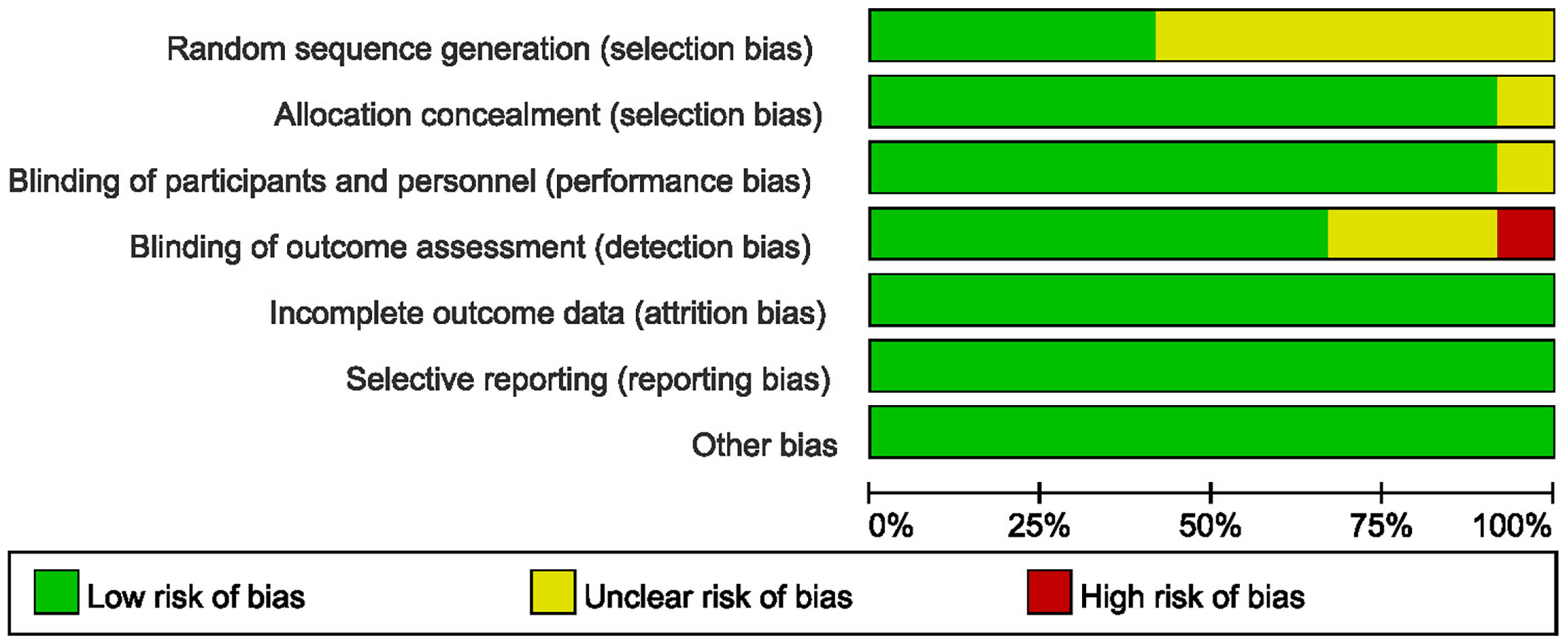
Risk of bias graph.

**Figure 3:**
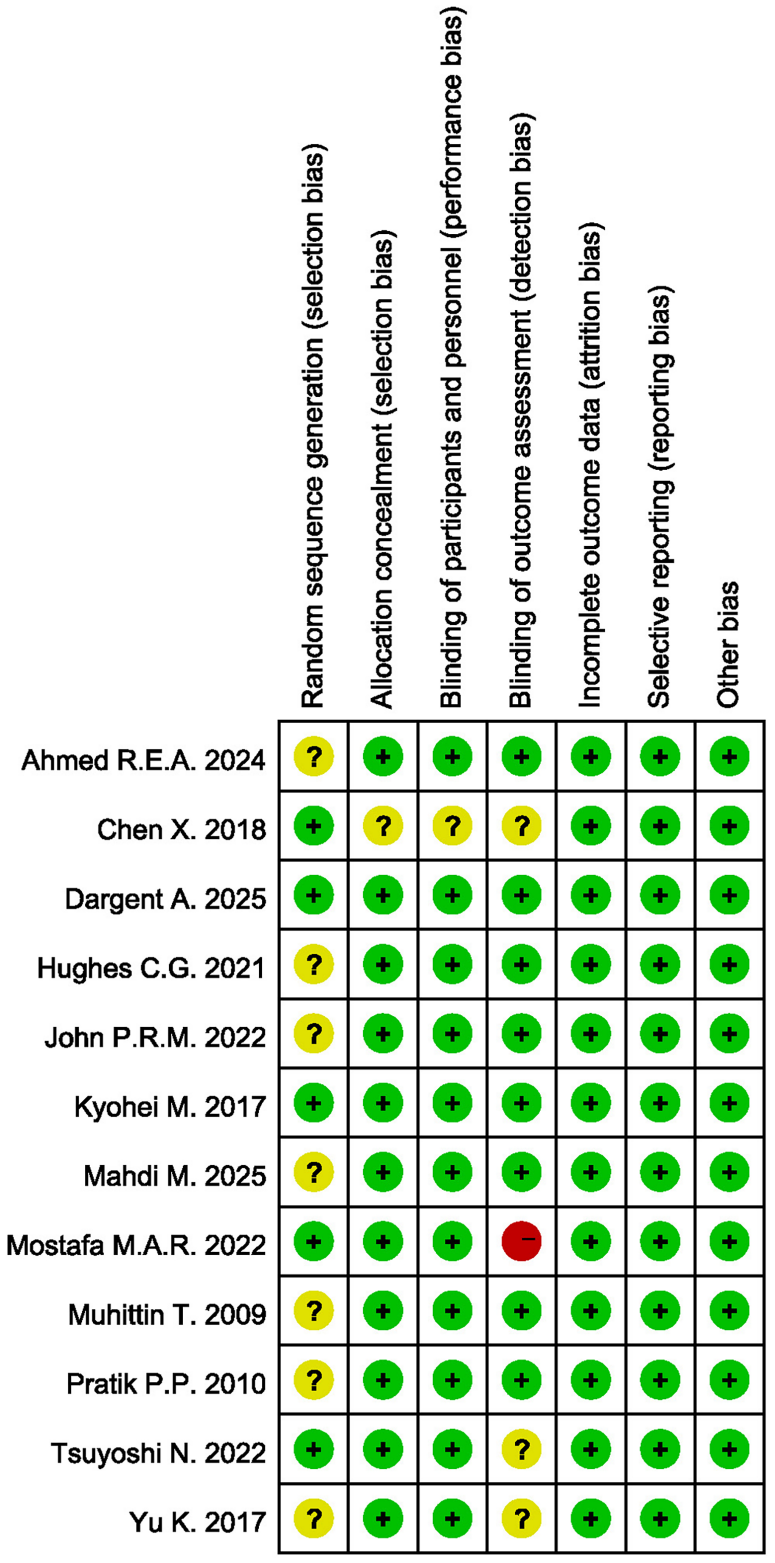
Risk of bias summary.

All included studies were assessed for quality across 7 domains: random sequence generation, allocation concealment, blinding of participants and personnel, blinding of outcome assessment, incomplete outcome data, selective reporting, and other biases (Figure 2). Among the studies included in this research, 1 study had 1 record of high risk of bias, 9 studies had 1 or more records of unclear risk of bias, and 2 studies had no records of either high or unclear risk of bias (Figure 3).

### 3.3 Primary outcomes

The peripheral blood inflammatory markers in patients with sepsis are the primary outcome measures of this study, including IL-6, TNF-*α*, and CRP. These markers directly reflect the inflammatory level and disease severity of sepsis patients, and are associated with the prognosis of the disease.

#### 3.3.1 Il-6

**Figure 4:**
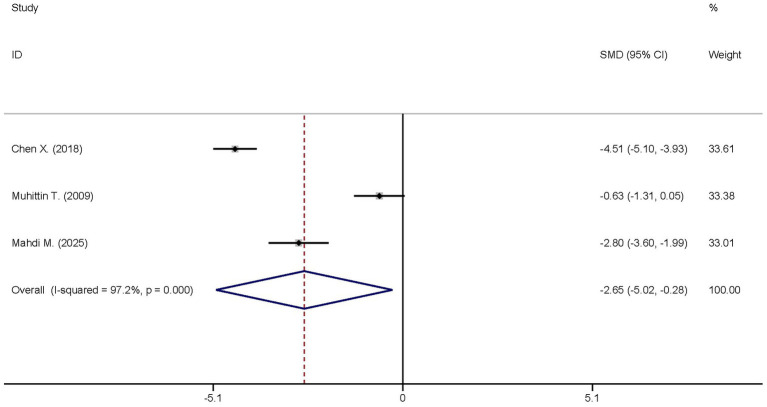
Forest plot of the IL-6. Model: Randomized model. Statistical method: Dersiminian-Larid (D-L) method. CI, confidence interval; SMD, standard mean difference.

Three studies reported the differences in the IL-6 levels between the dexmedetomidine treatment group and the non-dexmedetomidine treatment group. The dexmedetomidine treatment group included a total of 122 participants, while the non-dexmedetomidine treatment group had 121 participants. Heterogeneity analysis revealed significant heterogeneity among these studies (I^2^ = 97.2%, *p* < 0.001); therefore, a random-effects model was used to pool the effect sizes. Pooled analysis showed that dexmedetomidine reduced the peripheral blood IL-6 level in sepsis patients [standardized mean difference (SMD) = −2.65, 95% confidence interval (95%CI) = (−2.65, −0.28), *p* = 0.028], and this difference was statistically significant (Figure 4).

#### 3.3.2 TNF-*α*

**Figure 5:**
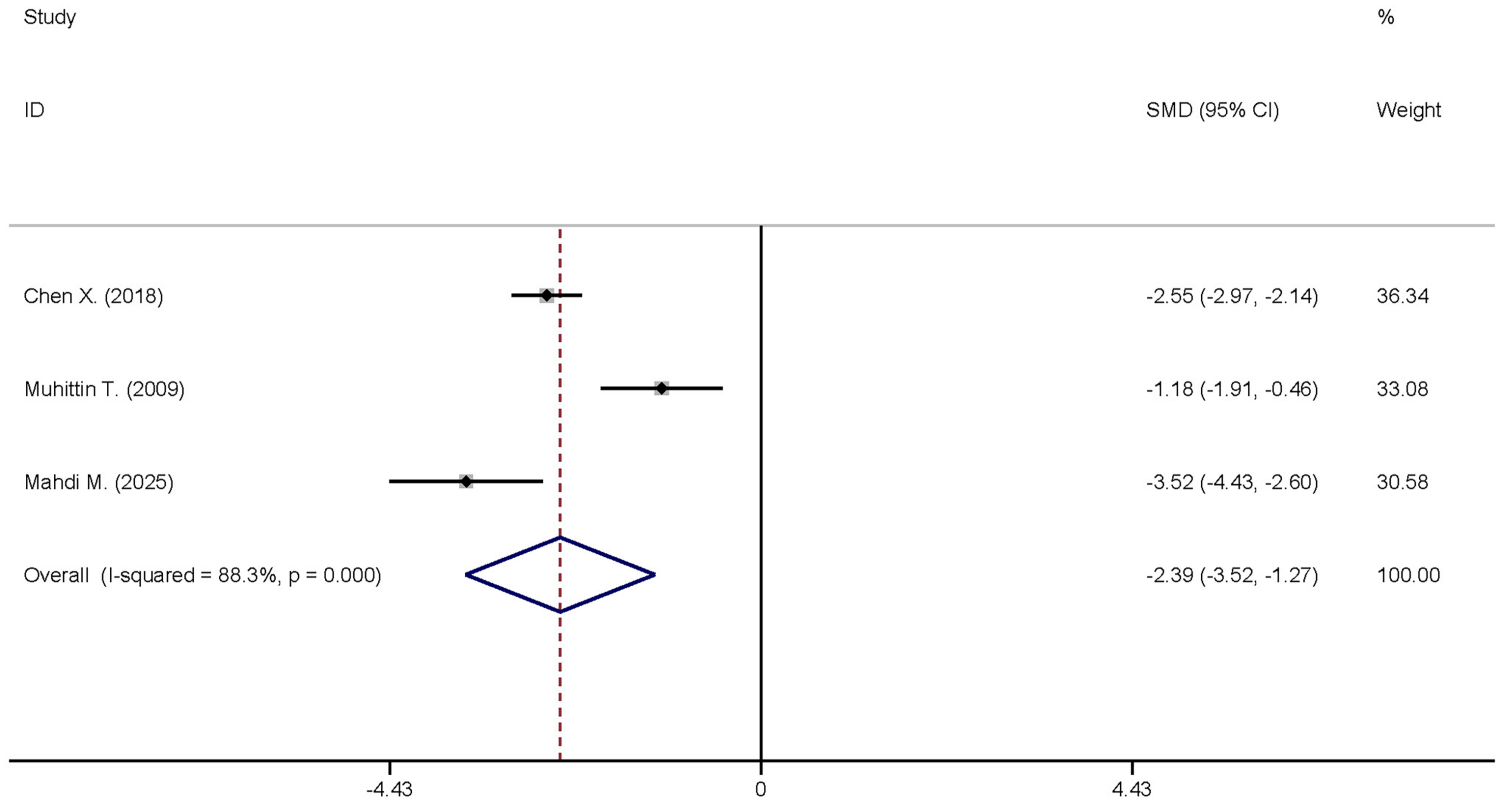
Forest plot of the TNF-α. Model: Randomized model. Statistical method: Dersiminian-Larid (D-L) method. CI, confidence interval; SMD, standard mean difference.

The same three studies analyzed the differences in the TNF-*α* levels between the dexmedetomidine treatment group and the non-dexmedetomidine treatment group. The dexmedetomidine treatment group comprised a total of 122 participants, and the non-dexmedetomidine treatment group had 121 participants. Heterogeneity analysis indicated significant heterogeneity among these studies (I^2^ = 88.3%, *p* < 0.001); thus, a random-effects model was applied to pool the effect sizes. Pooled analysis demonstrated that dexmedetomidine decreased the peripheral blood TNF-α level in sepsis patients [SMD = −2.39, 95%CI = (−3.52, −1.27), *p* < 0.001], with this difference being statistically significant (Figure 5).

#### 3.3.3 CRP

**Figure 6:**
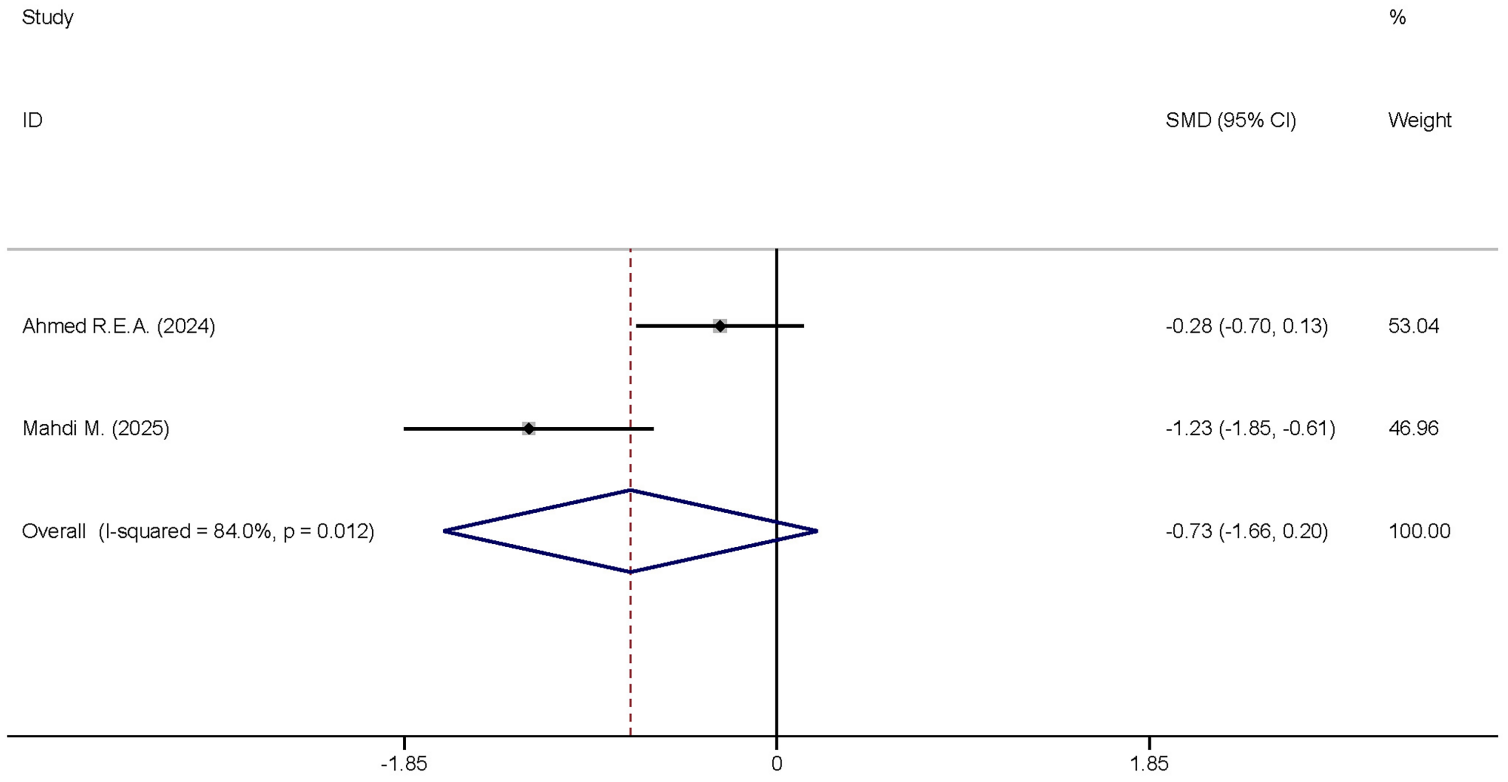
Forest plot of the CRP. Model: Fixed model. Statistical method: Inverse Variance (IV) method. CI, confidence interval; SMD, standard mean difference.

A total of two studies reported the differences in the CRP levels between the dexmedetomidine treatment group and the non-dexmedetomidine treatment group. The two groups had the same number of participants, with 69 individuals in each. Heterogeneity analysis revealed significant heterogeneity among these studies (I^2^ = 84.0%, *p* = 0.012); therefore, a random-effects model was used to pool the effect sizes. Pooled analysis showed that dexmedetomidine had no effect on the peripheral blood CRP level in sepsis patients [SMD = −0.0.73, 95%CI = (−1.66, 0.20), *p* = 0.125], and there was no statistically significant difference (Figure 6).

### 3.4 Secondary outcomes

To intuitively understand the clinical efficacy of dexmedetomidine in patients with sepsis, mortality, length of ICU stay, number of days free from mechanical ventilation on day 28, Sequential Organ Failure Assessment (SOFA) score, and Acute Physiology and Chronic Health Evaluation II (Apache II) score were included in this study as secondary outcome measures. Among these, mortality included in-hospital mortality, ICU mortality, and 28-day mortality.

#### 3.4.1 Hospital mortality

**Figure 7:**
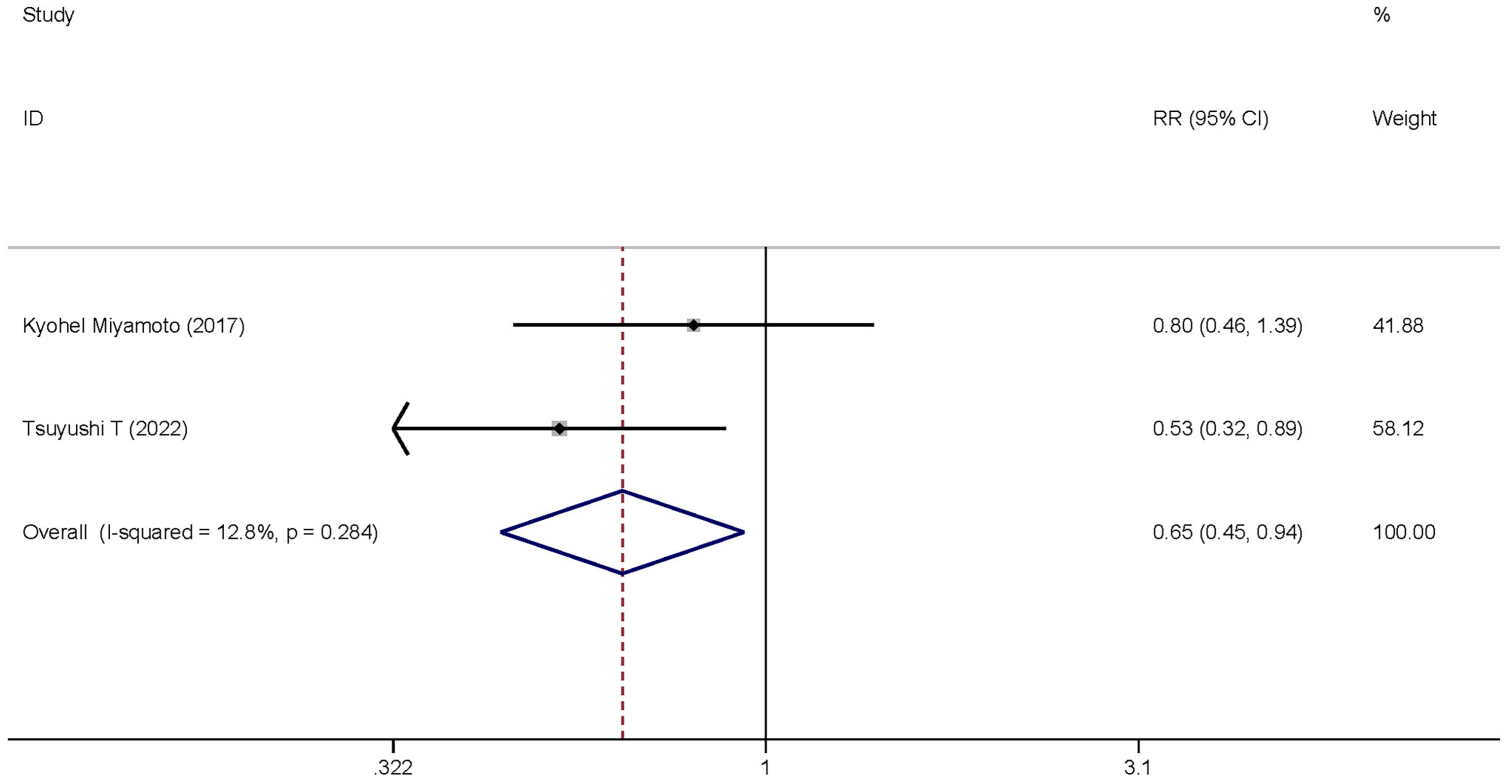
Forest plot of the hospital mortality. Model: Fixed model. Statistical method: Inverse Variance (IV) method. CI, confidence interval; RR, risk ratio.

Two studies investigated the differences in the hospital mortality between the dexmedetomidine treatment group and the non-dexmedetomidine treatment group. The dexmedetomidine treatment group had 114 participants, while the non-dexmedetomidine treatment group had 101 participants. Heterogeneity analysis showed no heterogeneity among these studies (I^2^ = 12.8%, *p* = 0.024); therefore, a fixed-effects model was used to pool the effect sizes. Pooled analysis indicated that dexmedetomidine reduced the in-hospital mortality of sepsis patients [relative risk (RR) = 0.65, 95%CI = (0.45, 0.94), *p* = 0.021], and this difference was statistically significant (Figure 7).

#### 3.4.2 ICU mortality

**Figure 8:**
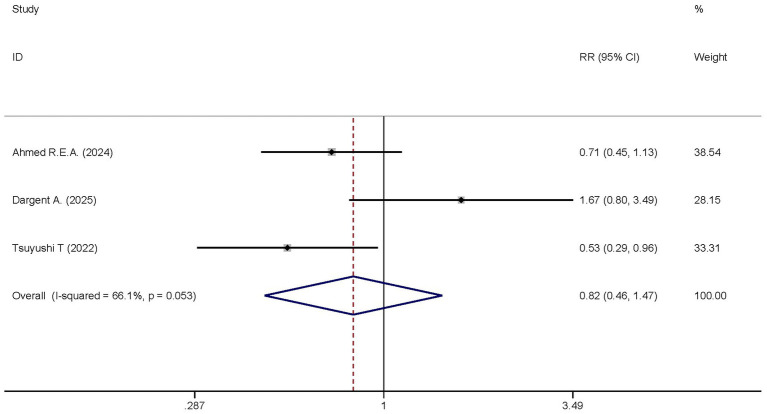
Forest plot of the ICU mortality. Model: Randomized model. Statistical method: Dersiminian-Larid (D-L) method. CI, confidence interval; RR, risk ratio.

Two studies reported the differences in ICU mortality between the dexmedetomidine treatment group and the non-dexmedetomidine treatment group. The dexmedetomidine treatment group included 115 participants, while the non-dexmedetomidine treatment group had 111 participants. Heterogeneity analysis revealed the presence of heterogeneity among these studies (I^2^ = 66.1%, *p* = 0.053); therefore, a random-effects model was used to pool the effect sizes. Pooled analysis showed that dexmedetomidine had no significant effect on the ICU mortality of sepsis patients [RR = 0.82, 95%CI = (0.46, 1.47), *p* = 0.501], and there was no statistically significant difference (Figure 8).

#### 3.4.3 28-day mortality

**Figure 9:**
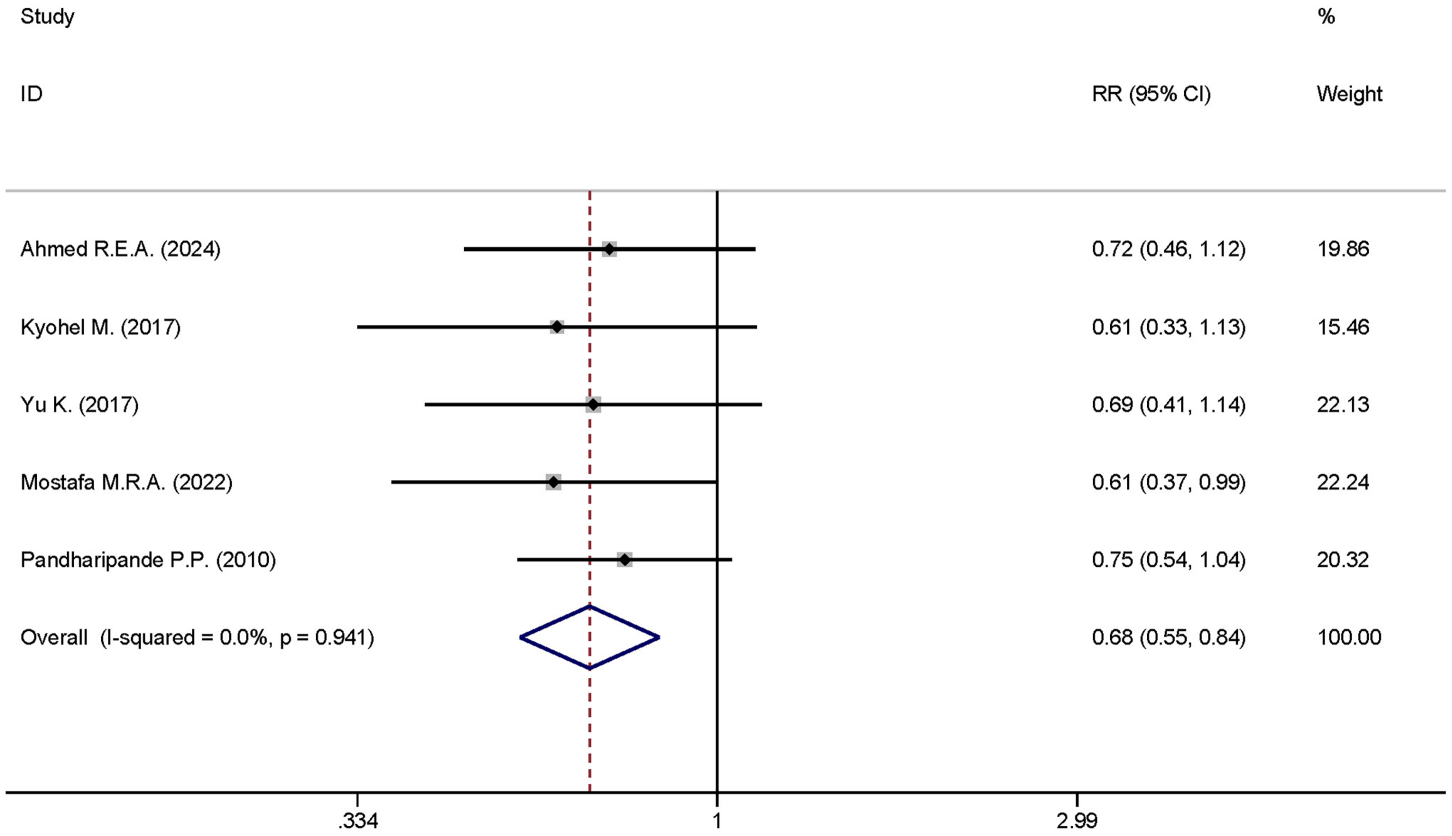
Forest plot of the mortality at day 28. Model: Randomized model. Statistical method: Dersiminian-Larid (D-L) method. CI, confidence interval; RR, risk ratio.

A total of five studies analyzed the differences in the 28-day mortality between the dexmedetomidine treatment group and the non-dexmedetomidine treatment group. The dexmedetomidine treatment group had 300 participants, while the non-dexmedetomidine treatment group included 293 participants. Heterogeneity analysis showed the presence of heterogeneity among these studies (I^2^ < 0.1%, *p* = 0.941); therefore, a fixed-effects model was used to pool the effect sizes. Pooled analysis demonstrated that dexmedetomidine significantly reduced the 28-day mortality of sepsis patients [RR = 0.68, 95%CI = (0.55, 0.84), *p* < 0.001], and this difference was statistically significant (Figure 9).

#### 3.4.4 Length of time in ICU stay

**Figure 10:**
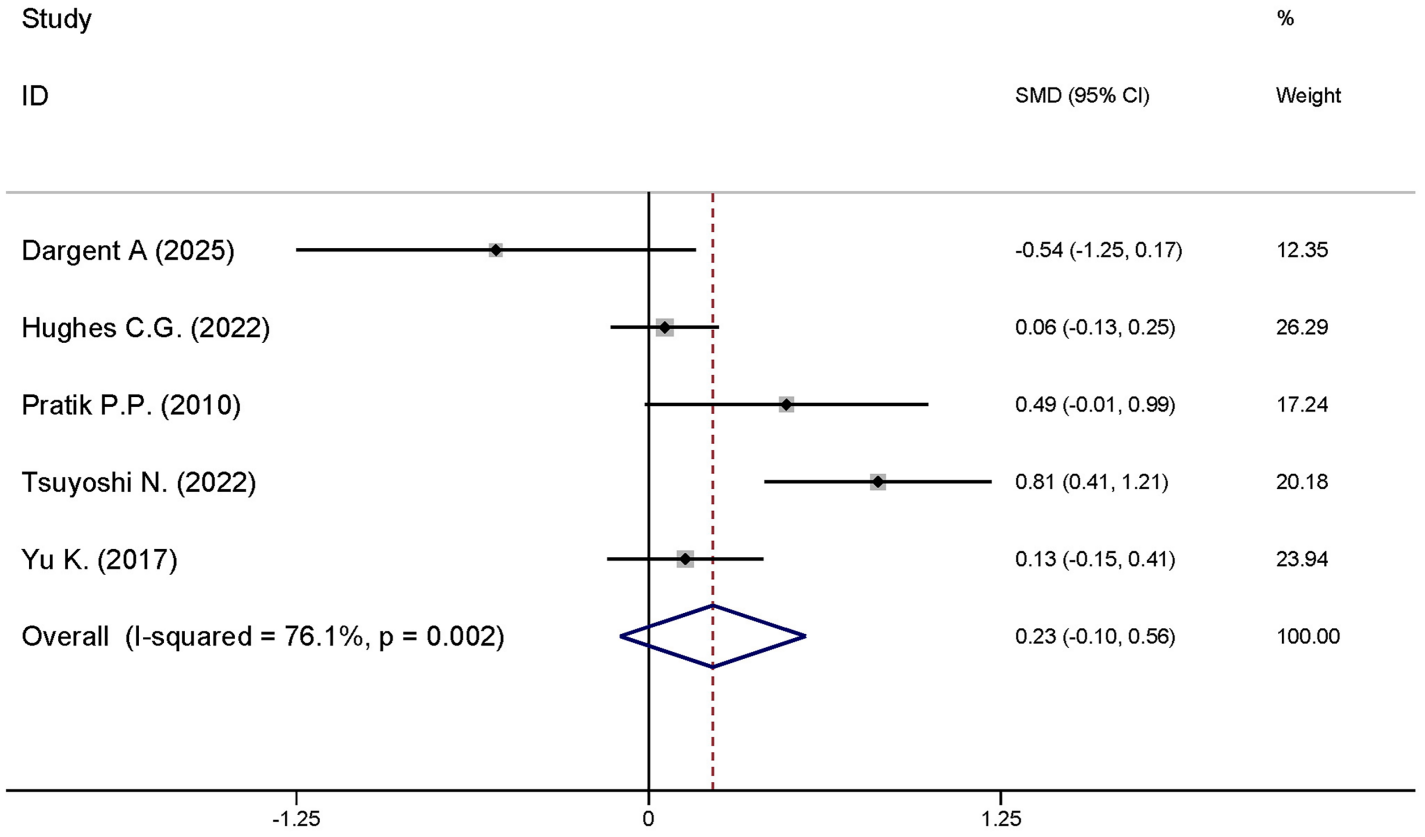
Forest plot of the ventilator-free days at day 28. Model: Fixed model. Statistical method: Inverse Variance (IV) method. CI, confidence interval; SMD, standard mean difference.

Six studies reported the differences in the length of ICU stay between the dexmedetomidine treatment group and the non-dexmedetomidine treatment group. The dexmedetomidine treatment group comprised 351 participants, while the non-dexmedetomidine treatment group had 345 participants. Heterogeneity analysis revealed the presence of heterogeneity among these studies (I^2^ = 26.7%, *p* = 0.234); therefore, a fixed-effects model was used to pool the effect sizes. Pooled analysis showed that dexmedetomidine had no effect on the length of ICU stay in sepsis patients [SMD = 0.04, 95%CI = (−0.11, 0.19), *p* = 0.574], and there was no statistically significant difference (Figure 10).

#### 3.4.5 Ventilator-free days at day 28

**Figure 11:**
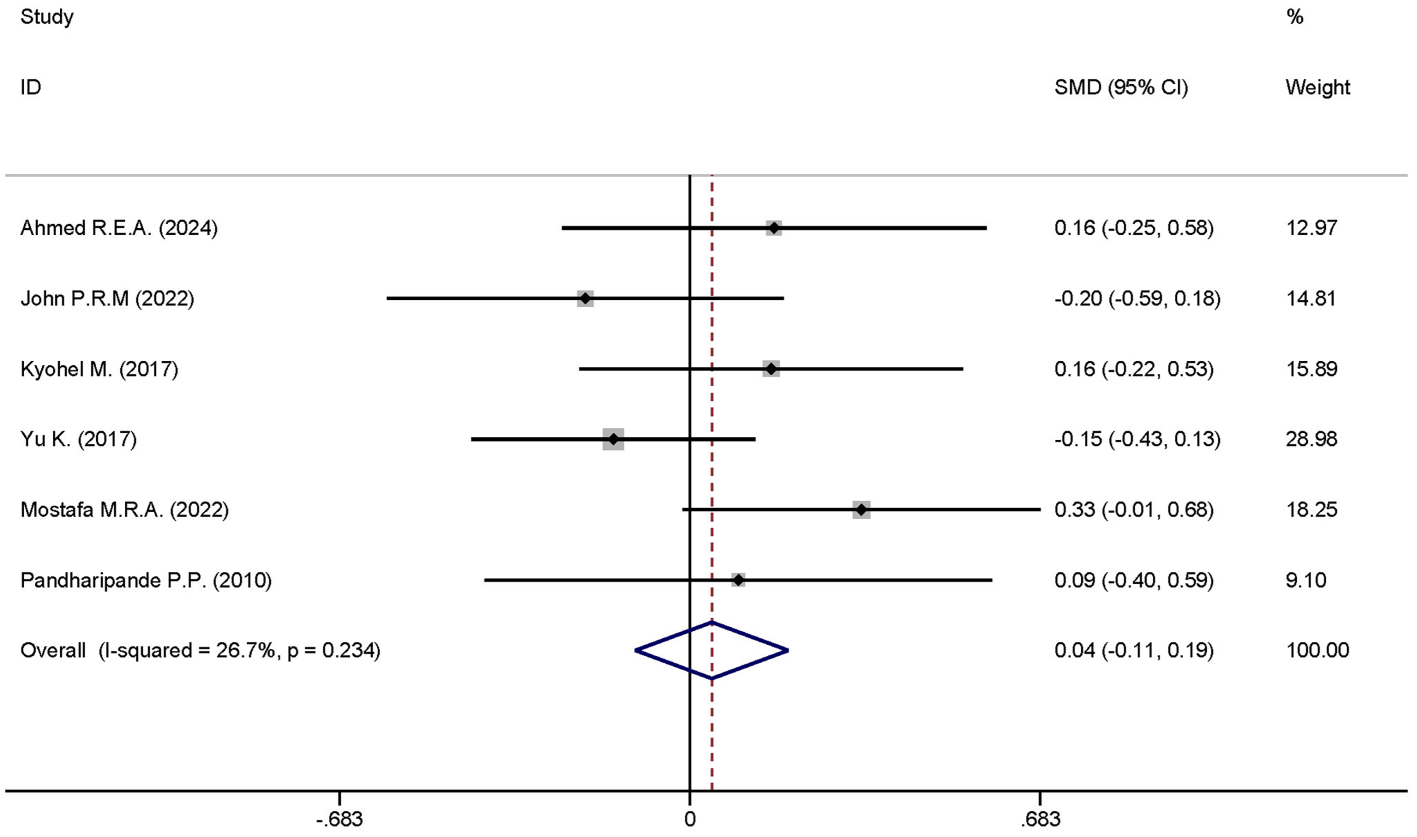
Forest plot of the length of hospital stay. Model: Fixed model. Statistical method: Inverse Variance (IV) method. CI, confidence interval; SMD, standard mean difference.

A total of five studies explored the differences in the ventilator-free days at day 28 between the dexmedetomidine treatment group and the non-dexmedetomidine treatment group. The dexmedetomidine treatment group had 351 participants, while the non-dexmedetomidine treatment group included 345 participants. Heterogeneity analysis revealed significant heterogeneity among these studies (I^2^ = 76.1%, *p* = 0.002); therefore, a random-effects model was used to pool the effect sizes. Pooled analysis showed that dexmedetomidine had no effect on ventilator-free days at day 28 in sepsis patients [SMD = 0.23, 95%CI = (−0.10, 0.56), *p* = 0.567], and there was no statistically significant difference (Figure 11).

#### 3.4.6 SOFA score

**Figure 12:**
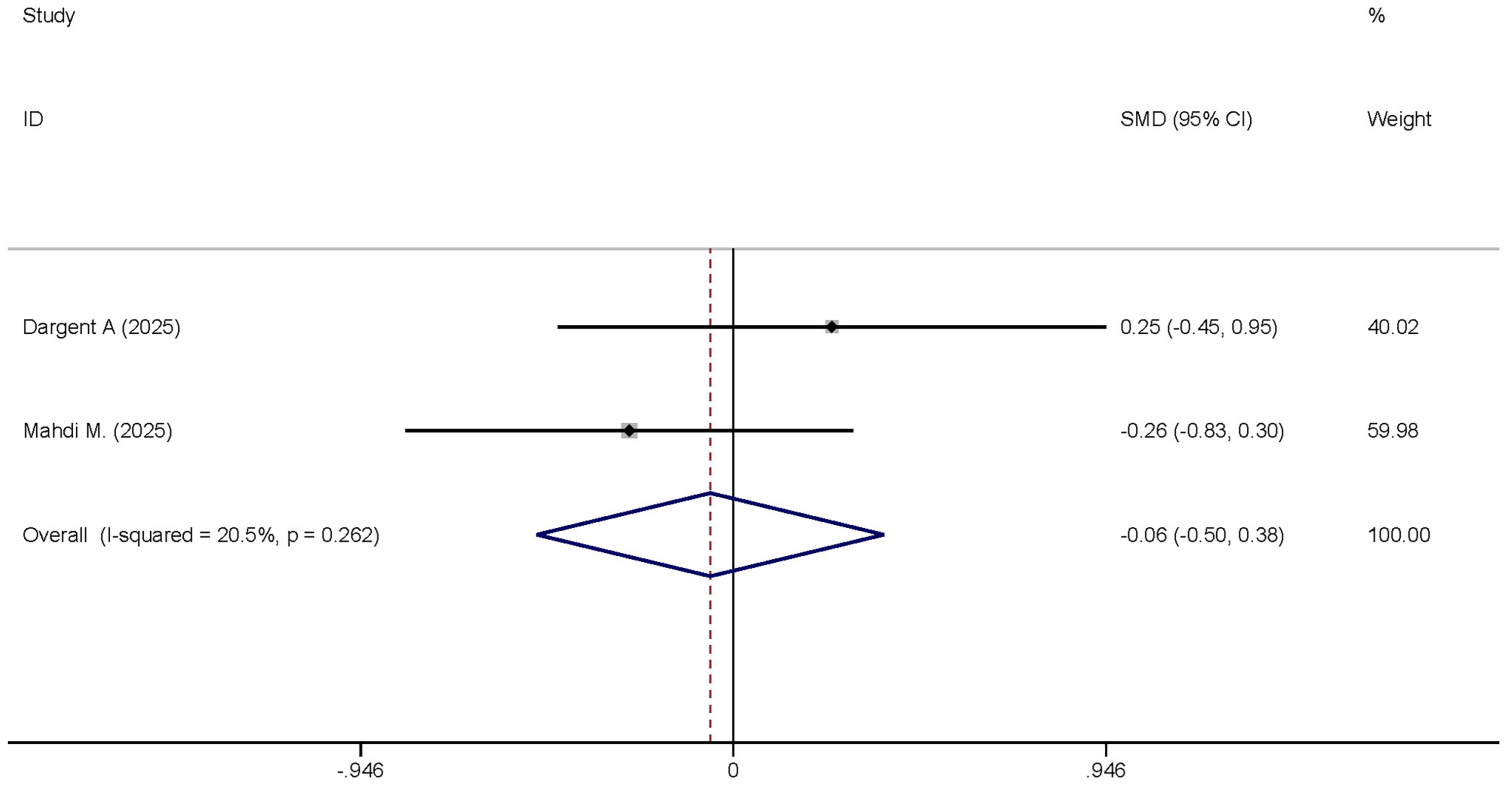
Forest plot of the SOFA score. Model: Fixed model. Statistical method: Inverse Variance (IV) method. CI, confidence interval; SMD, standard mean difference.

Two studies reported the differences in the SOFA scores between the dexmedetomidine treatment group and the non-dexmedetomidine treatment group. The dexmedetomidine treatment group had 40 participants, and the non-dexmedetomidine treatment group also had 40 participants. Heterogeneity analysis revealed the presence of heterogeneity among these studies (I^2^ = 20.5%, *p* = 0.262); therefore, a random-effects model was used to pool the effect sizes. Pooled analysis showed that dexmedetomidine had no effect on the SOFA scores of sepsis patients [SMD = −0.06, 95%CI = (−0.50, 0.38), *p* = 0.895], and there was no statistically significant difference (Figure 12).

#### 3.4.7 APACHE II scores

**Figure 13:**
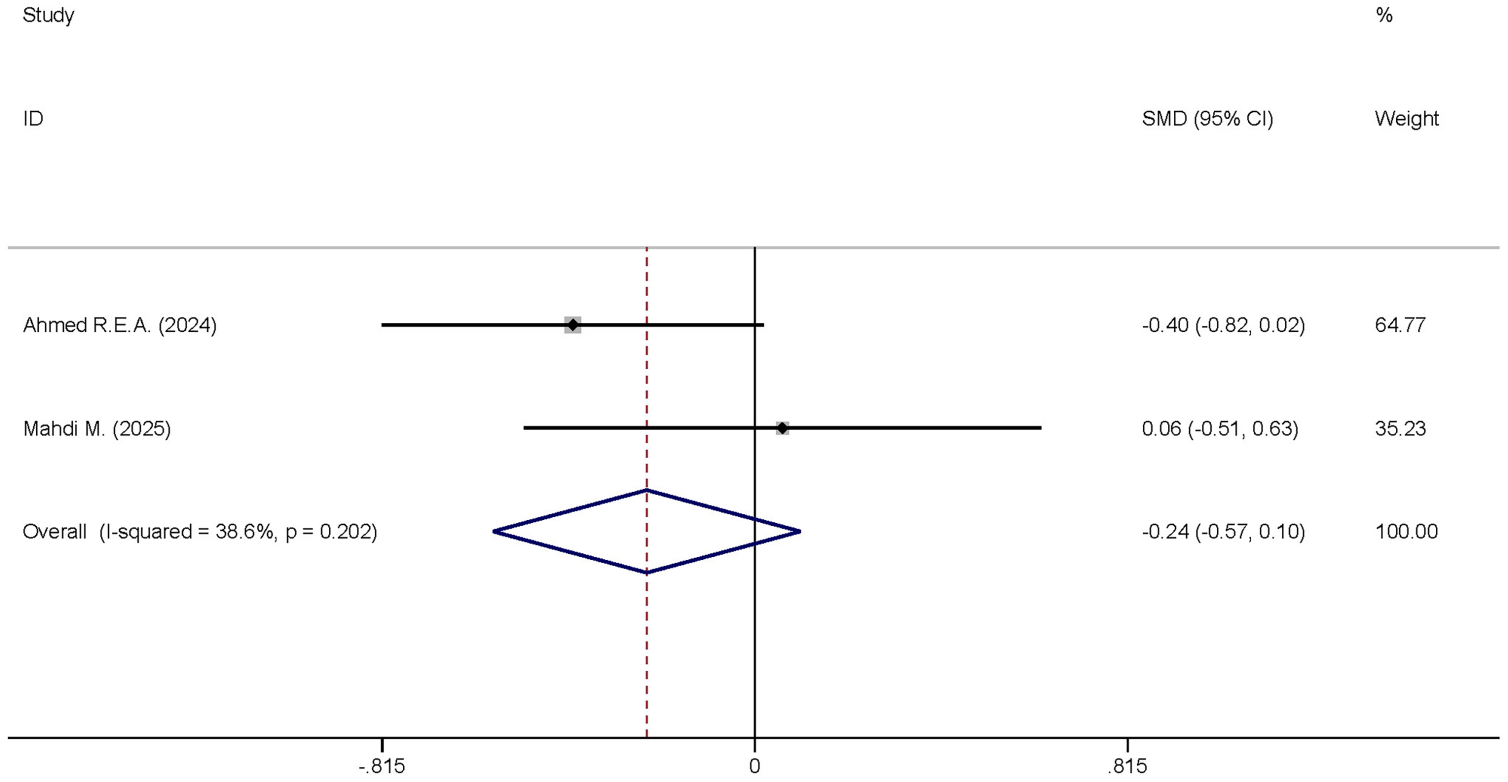
Forest plot of the Apache II score. Model: Fixed model. Statistical method: Inverse Variance (IV) method. CI, confidence interval; SMD, standard mean difference.

Two studies analyzed the differences in the APACHE II scores between the dexmedetomidine treatment group and the non-dexmedetomidine treatment group. The dexmedetomidine treatment group had 69 participants, and the non-dexmedetomidine treatment group also had 69 participants. Heterogeneity analysis revealed the presence of heterogeneity among these studies (I^2^ = 38.6%, *p* = 0.202); therefore, a random-effects model was used to pool the effect sizes. Pooled analysis showed that dexmedetomidine had no effect on the Apache II scores of sepsis patients [SMD = −0.24, 95%CI = (−0.57, 0.10), *p* = 0.168], and there was no statistical significance in this difference (Figure 13).

### 3.5 Sensitivity analysis

**Figure 14:**
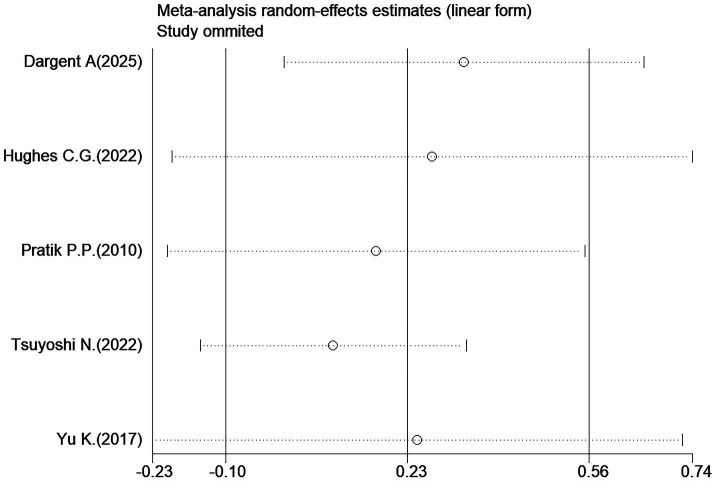
Sensitivity analysis of ventilator-free days at day 28.

**Figure 15:**
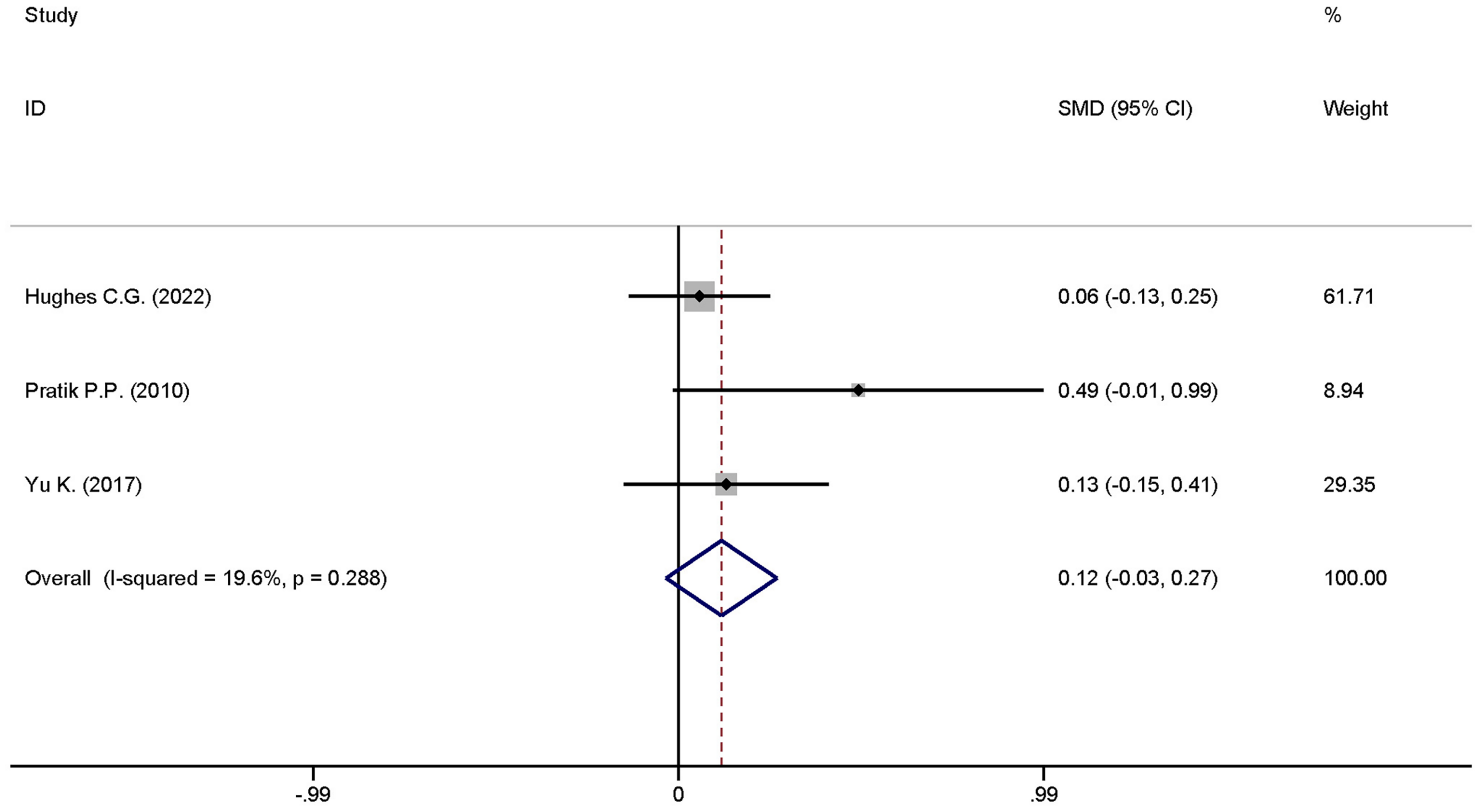
Forest plot of ventilator-free days at day 28 after sensitivity analysis adjustment. Model: Fixed model. Statistical method: Inverse Variance (IV) method. CI, confidence interval; SMD, standard mean difference.

Given that the outcome “ventilator-free days at day 28” included a large number of studies with significant heterogeneity, a sensitivity analysis was conducted to identify studies that significantly influenced the study results. The sensitivity analysis results showed that the studies by Dargent A. (22) and Nakashima N. (23) were the sources of significant heterogeneity (Figure 14). After excluding these two studies, the heterogeneity decreased; a re-analysis was performed using the adjusted fixed-effects model, and the results remained unchanged (Figure 15).

### 3.6 Publication bias

Sterne et al. (24) argued that when the number of included studies is less than 10, it is unnecessary to test the symmetry of the funnel plot (a tool for detecting publication bias) as the result would lack significance. In this study, the number of studies included for each outcome measure was less than 10; therefore, publication bias assessment was not applicable to this meta-analysis.

## 4 Discussion

Sepsis is a clinical syndrome characterized by heterogeneous pathophysiological mechanisms, primarily encompassing innate immune responses, acquired immune dysfunction, inflammatory cascades, procoagulant and antifibrinolytic processes, as well as alterations in cellular metabolism and signal transduction (25). Among these, inflammation serves as a pivotal link in the initiation and progression of sepsis. During the early phase of inflammation, the innate immune system is activated, releasing a large quantity of inflammatory factors that subsequently accumulate to form an “inflammatory storm,” which damages body tissues (26). After the peak of inflammation, the body’s immune system becomes suppressed, accompanied by massive depletion of lymphocytes and myeloid cells, and a significant reduction in inflammatory factors. At this stage, the body enters a state of low inflammatory levels, making it vulnerable to secondary infections by various pathogens (27). This meta-analysis demonstrated that dexmedetomidine exerts a positive effect on reducing interleukin-6 (IL-6) and tumor necrosis factor-*α* (TNF-α) levels in patients with sepsis, as well as lowering in-hospital mortality and 28-day mortality. However, dexmedetomidine shows no significant impact on C-reactive protein (CRP) levels, weaning duration, Acute Physiology and Chronic Health Evaluation II (APACHE II) score, Sequential Organ Failure Assessment (SOFA) score, length of ICU stay, or ICU mortality in these patients.

Tumor necrosis factor-α (TNF-α) is a pleiotropic proinflammatory cytokine that plays a central role in inflammation, cell apoptosis, and immune system regulation (28). Dajana F. Lendak and colleagues observed a significant increase in the levels of the TNF-α superfamily in sepsis patients, which was associated with the severity of sepsis and poor prognosis. This finding indicates that TNF-*α* exerts a crucial role in the occurrence and development of sepsis (29). Interleukin-6 (IL-6), a small-molecule polypeptide inflammatory cytokine, was first identified by Japanese scientists and exhibits a broad range of biological effects. IL-6 primarily participates in inflammatory responses, autoimmune reactions, and tumorigenesis by binding to the IL-6 receptor (30). In the early stage of acute infection, the elevation of IL-6 precedes that of C-reactive protein (CRP) and procalcitonin, and it shows a positive correlation with the body’s inflammatory level (31). Clinically, it is often used as an auxiliary indicator to assess the degree of inflammation and predict prognosis in patients. Interestingly, David Barrett found that IL-6 blockers demonstrated favorable efficacy in the treatment of cytokine storm syndrome (32). The results of our study showed that dexmedetomidine significantly reduced the levels of IL-6 and TNF-α in sepsis patients, while exerting no significant effect on CRP levels. This suggests that dexmedetomidine potentially modulates the production of early inflammatory mediators in sepsis, thereby slowing down the progression of further inflammatory cascades into an inflammatory storm. However, the specific biomolecular mechanisms underlying this process remain to be further investigated.

In 1999, the U.S. Food and Drug Administration (FDA) approved dexmedetomidine for intravenous sedation in ICU patients for the first time (33). To date, it has been used worldwide, including in Europe and China, with its indications gradually expanded. A growing body of studies have revealed that the effects of dexmedetomidine are not limited to sedation, analgesia, and anxiolysis. A previous study (34) comprehensively summarized the specific mechanisms by which dexmedetomidine protects vital organs in the human body by inhibiting ferroptosis. Dexmedetomidine also improves myocardial ischemia–reperfusion injury by inhibiting MDH2 lactation through regulating metabolic reprogramming (35). In addition, It has been found to exhibit potent anticancer effects in malignant tumors, including esophageal cancer, gastric adenocarcinoma, ovarian cancer, and osteosarcoma (36). Undoubtedly, dexmedetomidine is associated with several adverse reactions, particularly effects on hemodynamics. A large-sample randomized controlled trial (RCT) reported that bradycardia and hypotension were more common in mechanically ventilated ICU patients receiving dexmedetomidine compared with those receiving midazolam or propofol (37). Additionally, Singh et al. (38) documented cases of dexmedetomidine-related hypernatremia and polyuria. To directly evaluate the clinical benefits of dexmedetomidine in patients with sepsis, this study included the following as secondary outcome measures: length of ICU stay, ICU mortality, in-hospital mortality, 28-day mortality, weaning duration, Sequential Organ Failure Assessment (SOFA) score, and Acute Physiology and Chronic Health Evaluation II (APACHE II) score. Our results showed that dexmedetomidine significantly reduced in-hospital mortality and 28-day mortality in sepsis patients but had no impact on other clinical outcomes. We hypothesize that sepsis patients admitted to the ICU often have more complex conditions and are exposed to greater sensory stimuli (e.g., sound and light) and a higher risk of infection, which may prevent them from deriving benefits from dexmedetomidine. Therefore, early identification of sepsis and the use of dexmedetomidine in the early stage of sepsis to limit the progression of the inflammatory cytokine storm hold important practical significance.

This study systematically reviewed high-quality randomized controlled trials (RCTs) conducted previously across multiple countries and regions, which focused on dexmedetomidine intervention for patients with sepsis. The study boasts a high level of evidence and strong result credibility. Compared with previous studies, this research is more comprehensive, as it summarizes the clinical application value of dexmedetomidine in sepsis patients from three dimensions: inflammatory level, organ function status of the body, and clinical efficacy. Nevertheless, this study has certain limitations. Constrained by the reporting content and quantity of existing studies, this study was unable to determine the optimal dose and treatment course of dexmedetomidine. Additionally, there are discrepancies in the recommended maintenance doses of dexmedetomidine among different countries and regions. Finally, the potential molecular biological mechanisms underlying the reduction of IL-6 and TNF-*α* by dexmedetomidine require further investigation.

## 5 Conclusion

Dexmedetomidine can reduce the levels of IL-6 and TNF-αin patients with sepsis, while also decreasing in-hospital mortality and 28-day mortality. Furthermore, early identification of sepsis and subsequent administration of dexmedetomidine for sedation and anti-inflammatory therapy may yield more pronounced clinical benefits.

## Data Availability

The original contributions presented in the study are included in the article/supplementary material, further inquiries can be directed to the corresponding author/s.

## References

[1] Long B Gottlieb M. Emergency medicine updates: evaluation and diagnosis of sepsis and septic shock. Am J Emerg Med. (2025) 90:169–78. doi: 10.1016/j.ajem.2025.01.055, PMID: 39892181

[2] Rudd KE Johnson SC Agesa KM Shackelford KA Tsoi D Kievlan DR . Global, regional, and national sepsis incidence and mortality, 1990-2017: analysis for the global burden of disease study. Lancet. (2020) 395:200–11. doi: 10.1016/S0140-6736(19)32989-7, PMID: 31954465 PMC6970225

[3] Buchman TG Simpson SQ Sciarretta KL Finne KP Sowers N Collier M . Sepsis among Medicare beneficiaries: 1. The burdens of Sepsis, 2012-2018. Crit Care Med. (2020) 48:276–88. doi: 10.1097/CCM.0000000000004224, PMID: 32058366 PMC7017943

[4] Nelson LE Lu J Guo T Saper CB Franks NP Maze M. The alpha2-adrenoceptor agonist dexmedetomidine converges on an endogenous sleep-promoting pathway to exert its sedative effects. Anesthesiology. (2003) 98:428–36. doi: 10.1097/00000542-200302000-00024 12552203

[5] Keating GM. Dexmedetomidine: a review of its use for sedation in the intensive care setting. Drugs. (2015) 75:1119–30. doi: 10.1007/s40265-015-0419-5, PMID: 26063213

[6] Liu MW Zhang Y Xiong GF Zhang B-r Zhang Q-j Gao S-j . Dexmedetomidine for the treatment of sepsis-associated encephalopathy: mechanism and prospects. Biomed Pharmacother. (2025) 188:118209. doi: 10.1016/j.biopha.2025.118209, PMID: 40424824

[7] Xiao S Zhou Y Gao H Yang D. Dexmedetomidine attenuates airway inflammation and oxidative stress in asthma via the Nrf2 signaling pathway. Mol Med Rep. (2023) 27:2. doi: 10.3892/mmr.2022.12889, PMID: 36321783 PMC9673067

[8] Wang K Wu M Xu J Wu C Zhang B Wang G . Effects of dexmedetomidine on perioperative stress, inflammation, and immune function: systematic review and meta-analysis. Br J Anaesth. (2019) 123:777–94. doi: 10.1016/j.bja.2019.07.027, PMID: 31668347

[9] Srdić T Đurašević S Lakić I Ružičić A Vujović P Jevđović T . From molecular mechanisms to clinical therapy: understanding Sepsis-induced multiple organ dysfunction. Int J Mol Sci. (2024) 25:7770. doi: 10.3390/ijms25147770, PMID: 39063011 PMC11277140

[10] Kuang L Wu Y Shu J Yang J Zhou H Huang X. Pyroptotic macrophage-derived microvesicles accelerate formation of neutrophil extracellular traps via GSDMD-N-expressing mitochondrial transfer during sepsis. Int J Biol Sci. (2024) 20:733–50. doi: 10.7150/ijbs.87646, PMID: 38169726 PMC10758106

[11] Zhao X Xie J Duan C Wang L Si Y Liu S . ADAR1 protects pulmonary macrophages from sepsis-induced pyroptosis and lung injury through miR-21/A20 signaling. Int J Biol Sci. (2024) 20:464–85. doi: 10.7150/ijbs.86424, PMID: 38169584 PMC10758098

[12] She H Tan L Du Y Zhou Y Guo N Zhang J . VDAC2 malonylation participates in sepsis-induced myocardial dysfunction via mitochondrial-related ferroptosis. Int J Biol Sci. (2023) 19:3143–58. doi: 10.7150/ijbs.84613, PMID: 37416771 PMC10321281

[13] Qiao J Tan Y Liu H Yang B Zhang Q Liu Q . Histone H3K18 and Ezrin Lactylation promote renal dysfunction in Sepsis-associated acute kidney injury. Adv Sci. (2024) 11:e2307216. doi: 10.1002/advs.202307216, PMID: 38767134 PMC11267308

[14] Girard TD Ely EW. Delirium in septic patients: an unrecognized vital organ dysfunction In: Ortiz-Ruiz G Perafan MA Faist E Castell CD, editors. Sepsis. New York: Springer (2006). 136–50.

[15] Iten M Bachmann K Jakob SM Grandgirard D Leib SL Cioccari L. Adjunctive sedation with dexmedetomidine for the prevention of severe inflammation and septic encephalopathy: a pilot randomized controlled study. Crit Care Med. (2025) 53:e1377–88. doi: 10.1097/CCM.0000000000006655 40162868 PMC12203981

[16] Jiang L Hu M Lu Y Cao Y Chang Y Dai Z. The protective effects of dexmedetomidine on ischemic brain injury: a meta-analysis. J Clin Anesth. (2017) 40:25–32. doi: 10.1016/j.jclinane.2017.04.003 28625441

[17] Zhao Q Gong Z Wang J Fu L Zhang J Wang C . A zinc- and calcium-rich lysosomal Nanoreactor rescues monocyte/macrophage dysfunction under Sepsis. Adv Sci. (2023) 10:e2205097. doi: 10.1002/advs.202205097 PMC9951326 36596693

[18] Gong Y Hao W Xu L Yang Y Dong Z Pan P . BCG-derived outer membrane vesicles induce TLR2-dependent trained immunity to protect against Polymicrobial Sepsis. Adv Sci. (2025):e04101. doi: 10.1002/advs.202504101 PMC12499445 40552375

[19] Yuan HX Zhang LN Li G Qiao L. Brain protective effect of dexmedetomidine vs propofol for sedation during prolonged mechanical ventilation in non-brain injured patients. World J Psychiatry. (2024) 14:370–9. doi: 10.5498/wjp.v14.i3.370 38617978 PMC11008391

[20] Luo D Wan X Liu J Tong T. Optimally estimating the sample mean from the sample size, median, mid-range, and/or mid-quartile range. Stat Methods Med Res. (2018) 27:1785–805. doi: 10.1177/0962280216669183 27683581

[21] Wan X Wang W Liu J Tong T. Estimating the sample mean and standard deviation from the sample size, median, range and/or interquartile range. BMC Med Res Methodol. (2014) 14:135. doi: 10.1186/1471-2288-14-135 25524443 PMC4383202

[22] Dargent A Bourredjem A Jacquier M Bohe J Argaud L Levy B . Dexmedetomidine to reduce vasopressor resistance in Refractory septic shock: α2 agonist Dexmedetomidine for REfractory septic shock (ADRESS): a double-blind randomized controlled pilot trial. Crit Care Med. (2025) 53:e884–96. doi: 10.1097/CCM.0000000000006608 40019329 PMC11952692

[23] Nakashima T Miyamoto K Shima N Kato S Kawazoe Y Ohta Y . Dexmedetomidine improved renal function in patients with severe sepsis: an exploratory analysis of a randomized controlled trial. J Intensive Care. (2020) 8:1. doi: 10.1186/s40560-019-0415-z 31908779 PMC6939335

[24] Sterne JA Sutton AJ Ioannidis JP Terrin N Jones DR Lau J . Recommendations for examining and interpreting funnel plot asymmetry in meta-analyses of randomised controlled trials. BMJ. (2011) 343:d4002. doi: 10.1136/bmj.d4002 21784880

[25] O’Brien JM Ali NA Aberegg SK Abraham E. Sepsis. Am J Med. (2007) 120:1012–22. doi: 10.1016/j.amjmed.2007.01.035 18060918

[26] Delano MJ Ward PA. The immune system’s role in sepsis progression, resolution, and long-term outcome. Immunol Rev. (2016) 274:330–53. doi: 10.1111/imr.12499 27782333 PMC5111634

[27] Boomer JS Green JM Hotchkiss RS. The changing immune system in sepsis: is individualized immuno-modulatory therapy the answer? Virulence. (2014) 5:45–56. doi: 10.4161/viru.26516 24067565 PMC3916383

[28] Bradley JR. TNF-mediated inflammatory disease. J Pathol. (2008) 214:149–60. doi: 10.1002/path.2287 18161752

[29] Lendak DF Mihajlović DM Novakov-Mikić AS Mitić IM Boban JM Brkić SV. The role of TNF-α superfamily members in immunopathogenesis of sepsis. Cytokine. (2018) 111:125–30. doi: 10.1016/j.cyto.2018.08.015 30142533

[30] Tanaka T Narazaki M Kishimoto T. IL-6 in inflammation, immunity, and disease. Cold Spring Harb Perspect Biol. (2014) 6:a016295. doi: 10.1101/cshperspect.a016295 25190079 PMC4176007

[31] Espindola SL Fay J Carballo GM Pereson MJ Aloisi N Badano MN . Secondary dengue infection elicits earlier elevations in IL-6 and IL-10 levels. Int J Mol Sci. (2024) 25:11238. doi: 10.3390/ijms252011238 39457019 PMC11508614

[32] Barrett D. IL-6 Blockade in Cytokine Storm Syndromes. Adv Exp Med Biol. (2024) 1448:565–72. doi: 10.1007/978-3-031-59815-9_37 39117839

[33] Coursin DB Coursin DB Maccioli GA. Dexmedetomidine. Curr Opin Crit Care. (2001) 7:221–6. doi: 10.1097/00075198-200108000-00002 11571417

[34] Tian L Liu Q Wang X Chen S Li Y. Fighting ferroptosis: protective effects of dexmedetomidine on vital organ injuries. Life Sci. (2024) 354:122949. doi: 10.1016/j.lfs.2024.122949 39127318

[35] She H Hu Y Zhao G Du Y Wu Y Chen W . Dexmedetomidine ameliorates myocardial ischemia-reperfusion injury by inhibiting MDH2 Lactylation via regulating metabolic reprogramming. Adv Sci. (2024) 11:e2409499. doi: 10.1002/advs.202409499 PMC11672254 39467114

[36] Carnet Le Provost K Kepp O Kroemer G Bezu L. Trial watch: dexmedetomidine in cancer therapy. Onco Targets Ther. (2024) 13:2327143. doi: 10.1080/2162402X.2024.2327143 PMC10936656 38481729

[37] Ingebrigtson M Miller JT. Adverse hemodynamic effects of dexmedetomidine in critically ill elderly adults. J Pharm Pract. (2023) 36:1319–23. doi: 10.1177/08971900221110159 35730589

[38] Singh H Jani C Chioma SU Walker A Abdalla M Espina TDP. Dexmedetomidine-associated hypernatremia and polyuria. Am J Ther. (2022) 29:e596–9. doi: 10.1097/MJT.0000000000001383 34010160

[39] Ezz Al-Regal AR Ramzy EA Atia AAA Emara MM. Dexmedetomidine for reducing mortality in patients with septic shock: a randomized controlled trial (DecatSepsis). Chest. (2024) 166:1394–405. doi: 10.1016/j.chest.2024.06.3794 39004217

[40] Singer M Deutschman CS Seymour CW Shankar-Hari M Annane D Bauer M . The third international consensus definitions for Sepsis and septic shock (Sepsis-3). JAMA. (2016) 315:801–10. doi: 10.1001/jama.2016.0287 26903338 PMC4968574

[41] Chen X Hu J Zhang C Pan Y Tian D Kuang F . Effect and mechanism of dexmedetomidine on lungs in patients of sepsis complicated with acute respiratory distress syndrome. Zhonghua Wei Zhong Bing Ji Jiu Yi Xue. (2018) 30:151–5. doi: 10.3760/cma.j.issn.2095-4352.2018.02.011 29402365

[42] Hughes CG Mailloux PT Devlin JW Swan JT Sanders RD Anzueto A . Dexmedetomidine or Propofol for sedation in mechanically ventilated adults with Sepsis. N Engl J Med. (2021) 384:1424–36. doi: 10.1056/NEJMoa2024922 33528922 PMC8162695

[43] Moore JPR Shehabi Y Reade MC Bailey M Fraser JF Murray L . Stress response during early sedation with dexmedetomidine compared with usual-care in ventilated critically ill patients. Crit Care. (2022) 26:359. doi: 10.1186/s13054-022-04237-0 36419197 PMC9682690

[44] Miyamoto K Nakashima T Shima N Kato S Ueda K Kawazoe Y . Effect of Dexmedetomidine on lactate clearance in patients with septic shock: a subanalysis of a multicenter randomized controlled trial. Shock. (2018) 50:162–6. doi: 10.1097/SHK.0000000000001055 29117063

[45] Bone RC Balk RA Cerra FB Dellinger RP Fein AM Knaus WA . Definitions for sepsis and organ failure and guidelines for the use of innovative therapies in sepsis. The ACCP/SCCM consensus conference committee. American College of Chest Physicians/Society of Critical Care Medicine. America: Chest. (1992) 101:1644–55. doi: 10.1378/chest.101.6.1644 1303622

[46] Mokhlesian M Heydari F Boskabadi SJ Baradari AG Ajami A Alizadeh-Navaei R. The effect of dexmedetomidine on inflammatory factors and clinical outcomes in patients with septic shock: a randomized clinical trial. Clin Ther. (2025) 47:e9–e17. doi: 10.1016/j.clinthera.2024.11.004 39638724

[47] Mohamed ARM Laila AE Reem HE Amr FH. Effect of dexmedetomidine vs midazolam on the microcirculation of septic patients who are mechanically ventilated. Egypt J Anaesth. (2022) 38:459–65. doi: 10.1080/11101849.2022.2109826

[48] Tasdogan M Memis D Sut N Yuksel M. Results of a pilot study on the effects of propofol and dexmedetomidine on inflammatory responses and intraabdominal pressure in severe sepsis. J Clin Anesth. (2009) 21:394–400. doi: 10.1016/j.jclinane.2008.10.010 19833271

[49] Pandharipande PP Sanders RD Girard TD McGrane S Thompson JL Shintani AK . Effect of dexmedetomidine versus lorazepam on outcome in patients with sepsis: an a priori-designed analysis of the MENDS randomized controlled trial. Crit Care. (2010) 14:R38. doi: 10.1186/cc8916 20233428 PMC2887145

[50] Kawazoe Y Miyamoto K Morimoto T Yamamoto T Fuke A Hashimoto A . Effect of Dexmedetomidine on mortality and ventilator-free days in patients requiring mechanical ventilation with Sepsis: a randomized clinical trial. JAMA. (2017) 317:1321–8. doi: 10.1001/jama.2017.2088 28322414 PMC5469298

